# Account of Two Cases of Tic Douloureux, Cured by the External Use of the Atropa Belladonna

**Published:** 1825-06

**Authors:** 

**Affiliations:** Assistant-Surgeon, 66th Regiment.


Art. V.—*Account of two Cases of Tic Douloureux, cured by the external Use of the Atropa Belladonna.* By — Henry, Esq. Assistant-Surgeon, 66th Regiment.

Case I.—Serjeant John Gale, hospital-serjeant, 66th regiment, thirty-nine years of age, delicate, subject to inflammatory attacks, went to bed on the night of the 2d of March, 1824, quite well. He awoke with a strange pulsation and tingling over the right eyebrow, which soon increased to a sensation of heat and soreness, and at length to a most excruciating pain, extending across the temple and shooting down the cheek. This was attended with an involuntary constriction of the eyeball, and a copious effusion of tears. The focus of the pain appeared to be the supra orbitary foramen of the os frontis. The paroxysm lasted two or three minutes, when there was an interval of partial, and sometimes complete, ease, for five, six, or ten minutes; then the same symptoms recommenced, and, after four or five hours of alternate torture and comparative happiness, the disease ceased, having worn itself out. The forehead and temple remained numb, and tender to the touch, the day after.

The night of the 3d March was passed by the patient in the same way as the former, and the paroxysms were even more severe.

Dr. Burton, surgeon 66th regiment, and the writer, attended the man. From a belief that the disease was in some way connected with vascular fulness in the system, as well as topical congestion, (the man's face being very florid,) the depletory plan was carried into effect. Blood in considerable quantities was abstracted, generally and locally; the bowels were briskly purged; blisters were put on the right temple, and behind the ear. The patient was confined to bed, and kept on spoon-diet; and all sources of nervous irritation were carefully avoided.

Notwithstanding that this method of cure, (apparently so plainly indicated by the symptoms,) was carefully carried into effect, under the superintendence of Dr. Burton, the patient derived very little advantage from it. The attack came on regularly every night; his health was suffering much; and at length we began to think of dividing and removing a portion of the frontal filament of the ophthalmic branch of the fifth pair of nerves, where, without doubt, the disease was seated. The paroxysms had neither been reduced in frequency nor moderated in violence.

In the mean time Dr. Burton became ill, and confined to bed, when the care of the patient devolved on the writer. In reading cases of tic douloureux, it had often occurred to him that some powerful sedative medicine, concentrated and directed to the seat of the pain, might be useful; and, by the analogy of the direct influence of the atropa belladonna over the iris, he inferred that it might be that medicine. It was probable that the same subtle and powerful effluvium, which, through an intricate and circuitous channel, could contract the fibres of the iris, through the medium of its nerves, might also control the morbid action of a nerve lying almost in contact with it, or produce at least some change which would be beneficial. The very circumstance of the name of the belladonna seemed to point out its peculiar virtues in diseases connected with the skin; since it was so called by the Italians, from its use as a cosmetic amongst the ladies of Italy.

The writer moistened about ten grains of the extract with a few drops of water, and, during a violent paroxysm, rubbed the paste over the eyebrow for about three minutes, as near as he could judge, where the frontal nerve emerges. *The result was an instantaneous abatement of the pain.* It returned, however, in half an hour; but on that night (which was the twelfth from the first attack,) the patient slept better, and had fewer and shorter fits of pain, than on any preceding one since the commencement of his illness. The same application was repeated on the next night, with similar good effect. On the fourteenth night there was an intermission. On the fifteenth the medicine was again used, and the patient passed the night tolerably well. The disease continued to yield; and, finally, in three weeks the man was quite free from tic douloureux.

It must not be concealed, however, that, since the above period, Serjeant Gale has had two or three twitches in the same nerve, but not productive of any serious inconvenience or pain. So convinced is he of the good effect of the belladonna, that, on these occasions, he has had recourse to it of his own accord; and he states that it has always relieved the pain in his eyebrow.

Case II.—Mrs. Margaret M'Kim, a lady residing at Ballimar, within a mile of Sligo, fifty years of age, plethoric, but healthy, was attacked with a sensation of creeping and tingling over the right eyebrow, on the evening of the 26th March last, which gradually changed into a convulsive movement of the levator palpebræ and orbicular muscles, with lancinating pain over the brow, radiating from it across the temple and down the cheek, up the forehead, and into the orbit of the right side of the face. The acuteness of the pain continued only three or four minutes, —was followed by about a quarter of an hour's ease, and then came on as bad as ever. It was so extremely distressing, that during seven nights sleep was altogether banished; and the patient was quite miserable during the day, in the anticipation of what awaited her at night. The succession of paroxysms constituting (as it may be termed) the *fit* of the disease, lasted about six hours; and in this, as well as in the case of Serjeant Gale, an unpleasant feeling of numbness, and slight irregular twitches of the muscles of the eyelids, were left behind.

On the 6th instant, the writer was requested to see this lady. As no medicine had been taken, and, with the exception of fomentations, no external application used, the case was considered a favourable one for the trial of the power of the belladonna. The writer told Mrs. M'Kim that he had used a medicine once in a case similar to hers, and would try its virtue again on her disease, if she had no objection; adding that, very probably, it would act like a charm in soothing and removing the pain. She consented immediately. About the size of a pea of the extract was moistened with a little water, and rubbed with the finger over the seat of the most excruciating pain at the supra-orbital foramen. The friction was continued about five minutes, when the iris of the right eye became considerably expanded; it was then discontinued. In less than ten minutes the lady exclaimed, "I feel no pain!" About five minutes more elapsed, when the convulsive twitchings of the muscles of the eyelids also ceased; and from that hour till the present the patient has not had the slightest return of the disease, and is now in excellent health.

The writer is not aware that this valuable medicine has ever been used externally for the cure of tic douloureux before.* The leaves of the plant have been frequently employed in discussing indolent scrofulous and schirrous tumors; and, in a late medical publication, they have been recommended as useful in obstinate chronic rheumatism. It is quite possible that, in the wide field of foreign and domestic medical literature, (to *all* parts of which, from local circumstances, access is denied the writer,) the above-mentioned virtues of the belladonna may have been noticed and recorded; but its employment by him was solely attributable to the consideration of its direct influence over the nerves, as mentioned in the case of Serjeant Gale.

---

* A case of tic douloureux, cured by the external use of the extract of belladonna, was published, some years ago, in this Journal, by one of the present Editors.—Edit.

It is likely that the publicity which the cure last effected has attained in this neighbourhood, may procure the writer another opportunity of using the belladonna. It is his intention, in the event of this occurring, to use the extract of the datura stramonium, (which also possesses the power of dilating the iris,) and to compare its virtues, if it have any, with those of the belladonna.

*Sligo; April 17th,* 1825.

